# Liver abscess caused by fish bone perforation of Meckel’s diverticulum: a case report

**DOI:** 10.1186/s12893-020-00783-y

**Published:** 2020-06-05

**Authors:** Seiji Natsuki, Yasuhito Iseki, Hishashi Nagahara, Tatsunari Fukuoka, Masatsune Shibutani, Masaichi Ohira

**Affiliations:** Department of Gastroenterological surgery, Osaka City University Graduate School of Medicine, 1-5-7 asahimachi, abeno-ku, osaka-shi, Osaka-fu, 545-8586 Japan

**Keywords:** Liver abscess, Fish bone, Foreign body perforation, Meckel’s diverticulum

## Abstract

**Background:**

Liver abscess due to gastrointestinal perforation by foreign bodies is rare. Furthermore, there are few case reports of liver abscess via the portal vein caused by perforation of the lower gastrointestinal tract by a foreign body.

**Case presentation:**

A 54-year-old man visited our hospital because of a fever that had lasted for 1 month. There were no physical findings except for the fever. Laboratory tests showed only elevated inflammatory markers. Abdominal contrast-enhanced computed tomography revealed an abscess in the right lobe of the liver and a high-density object in the small intestine. We diagnosed him with liver abscess secondary to intestinal perforation by a foreign body. The patient underwent drainage of the liver abscess and laparoscopic surgery for perforation of the small intestine. A fish bone had perforated the top of Meckel’s diverticulum, which had been covered by the ileal mesentery. We successfully performed diverticulectomy and removed the fish bone. The patient was discharged without complications on the 13th postoperative day.

**Conclusions:**

Liver abscess caused by foreign bodies requires multidisciplinary treatment, so we must detect and remove the cause of the abscess earlier. Liver abscess can form via the portal vein secondary to lower gastrointestinal perforation, as in this case. When exploring the cause of liver abscess, we should investigate the whole body, including the lower gastrointestinal tract.

## Background

Foreign bodies in the gastrointestinal (GI) tract are often encountered in clinical practice. Most ingested foreign bodies pass through uneventfully, and the incidence of intestinal perforation after foreign body ingestion is approximately 1% [1]. Foreign body perforation usually occurs due to fishbones, chicken bones, toothpicks, needles, or pens [2]. In Japan, foreign body perforation is often caused by fish bones [3]. Reported etiologies of fish bone perforation include (1) ingestion of a fish bone longer than the luminal diameter, (2) dysmotility due to abdominal adhesions, and (3) diverticula or hernia [4].

The most common etiologies of liver abscess include (1) complications of cholangitis, (2) bloodstream dissemination via the portal vein and hepatic artery in systemic sepsis, (3) local spread from infected contiguous tissue, and (4) traumatic injury [5, 6]. Liver abscess secondary to foreign body perforation is extremely rare [7, 8]. There have been several case reports of liver abscess due to foreign body in the GI tract penetrating the liver directly. There are also a few case reports of liver abscess via the portal vein caused by perforation of the lower GI tract by a foreign body.

We herein report a case of liver abscess secondary to Meckel’s diverticulum perforation induced by a fish bone.

## Case presentation

**Fig. 1:**
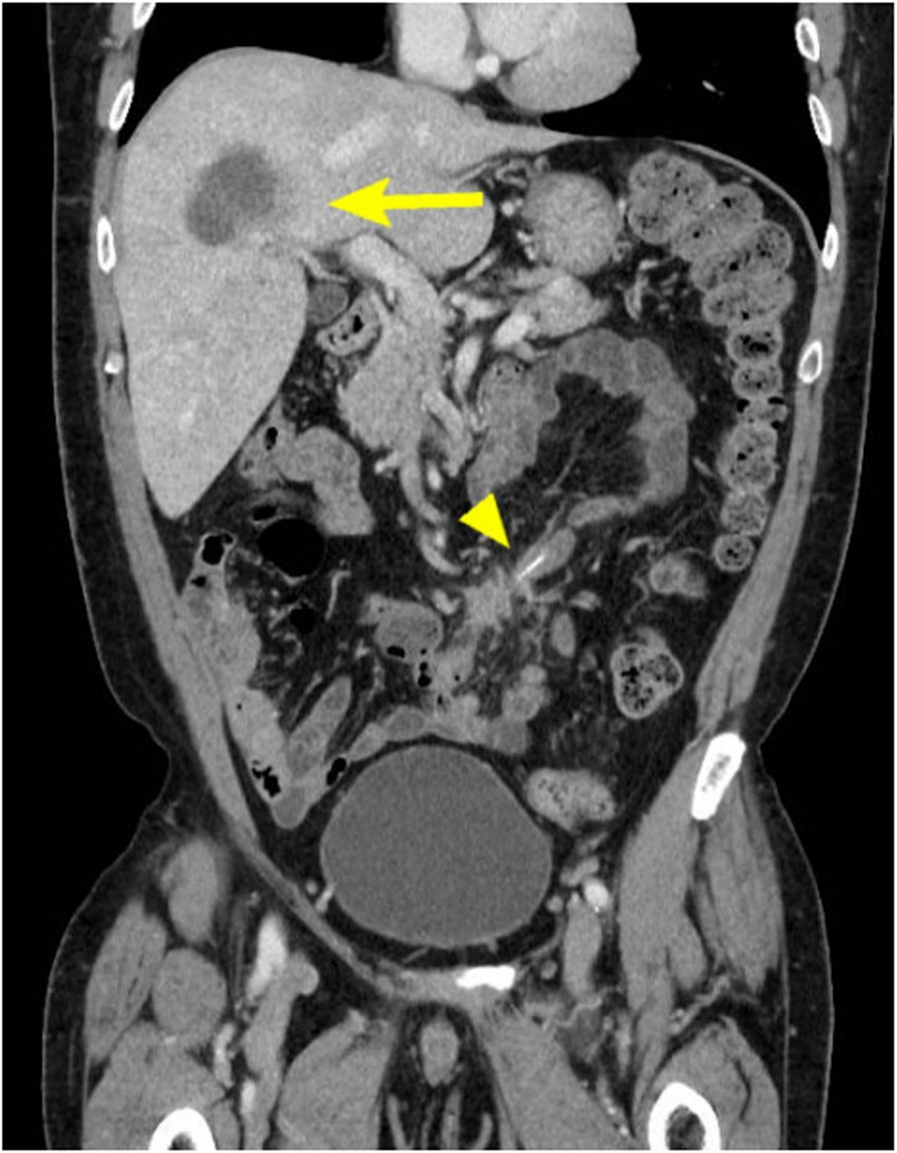
Enhanced computed tomography (CT) revealed an abscess in the right lobe of the liver and a needle-like foreign body in the small intestine with fat stranding

A 54-year-old man visited his primary doctor with a fever. He was prescribed common cold medicine, but his fever persisted. After visiting several hospitals, he was referred to the department of general practice at our hospital due to a 1-month history of a spiking fever. He had a medical history of hypertension. On admission, his body temperature was 37.5 °C. The findings from a physical examination were unremarkable, and there was no abdominal pain, nausea, or abdominal distension. The white blood cell count was 17,600/μL (80.5% neutrophils), and the C-reactive protein level was 7.66 mg/dL. Serum bilirubin, aspartate aminotransferase, and alanine aminotransferase levels were within normal range. Chest and abdominal X-ray findings were unremarkable; however, abdominal contrast-enhanced computed tomography (CT) revealed an abscess in the right lobe of the liver (segment 8) and a high-density linear object in the small intestine with fat stranding. There were no free air and other abdominal abscess (Fig. 1). On admission, blood cultures grew *Streptococcus oralis*.

The general practitioner suspected this to be a case of fish bone bowel perforation with liver abscess, and a surgical consult was thus requested. The patient often ate fish but did not recall whether or not he had swallowed any fish bones. The liver abscess was about 5 cm in size, so we drained it by a percutaneous approach and administered ceftriaxone and metronidazole. Purulent fluid cultures from the liver abscess grew *Streptococcus intermedius*. He had no abdominal symptoms, so he underwent double-balloon endoscopy (DBE) before surgery. We observed the lower ileum with DBE and the intestine downstream from the ileal end by X-ray fluoroscopy, but we were unable to detect any foreign bodies, diverticula, or leakage.

**Fig. 2:**
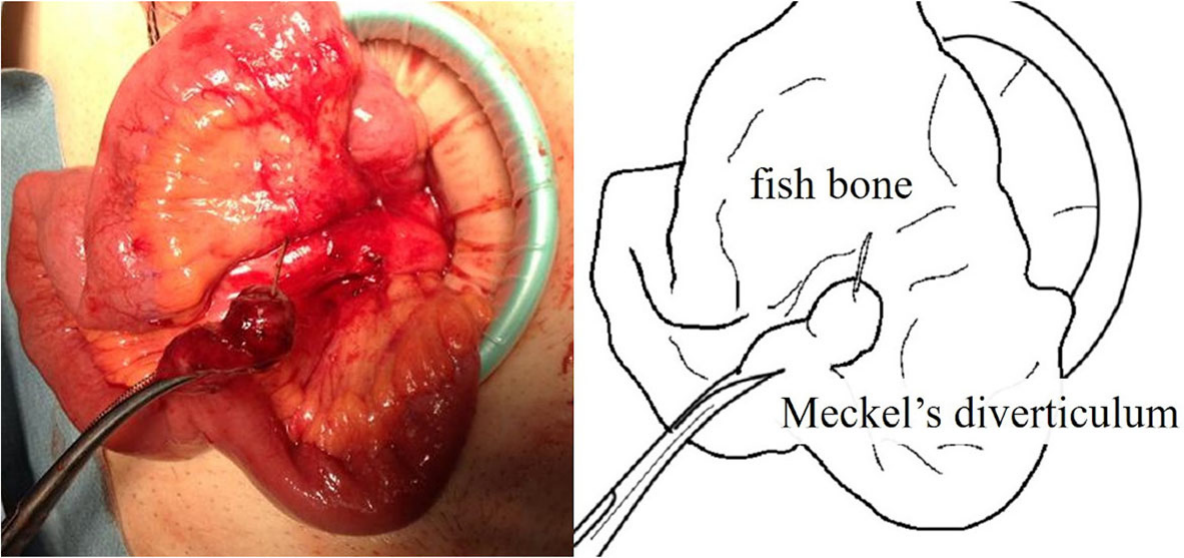
There was a fish bone perforating the top of the Meckel’s diverticulum, which had been covered by mesentery

We subsequently performed laparoscopic surgery due to suspicion of fish bone perforation. Laparoscopy showed that there was no ascites or abdominal abscess. Meckel’s diverticulum was located in the ileum approximately 50 cm upstream from the ileal end and had adhered to the ileal mesentery with inflammation. On peeling away the adhesion, we found a 2-cm fish bone perforating the top of Meckel’s diverticulum that had been covered by mesentery. There were no other abnormal findings of the intestine (Fig. 2). We thus performed diverticulectomy. In addition, we did not need to drain the liver abscess laparoscopically.

The histopathological report showed Meckel’s diverticulum without ectopic tissues. Recovery after the surgery was uneventful, and he was discharged on the 13th postoperative day.

## Discussion and conclusions

Most ingested foreign bodies are excreted without injuring the GI tract, and only 1% of people who ingest foreign bodies experience any symptoms, such as perforation [1]. The most common sites of perforation by foreign bodies are, in descending order, the ileum, transverse colon, and sigmoid colon [9]. The present patient had Meckel’s diverticulum presenting with fish bone perforation. Meckel’s diverticulum is the most common congenital malformation of the GI tract, occurring in 2–4% of the population [10]. Most patients with Meckel’s diverticulum are asymptomatic, but a few may develop GI bleeding, abdominal pain, bowel obstruction, or perforation. Proposed causes of Meckel’s diverticulum perforation include ectopic mucosa, a foreign body, inflammatory bowel disease (IBD), and tumors [11]. There have been several reports of Meckel’s diverticulum perforation due to a fish bone; however, almost all cases developed peritonitis and underwent surgery within a few days. In our case, the Meckel’s diverticulum with perforation induced by a fish bone had become covered by the mesentery, leading to localized inflammation. We therefore believe that our patient developed liver abscess secondarily after the symptom had persisted for 1 month.

Our patient had no abdominal symptoms, and we suspected that the ingested foreign body might have been able to be removed by endoscopy. For that reason, we performed DBE. However, we were unable to detect any lesions via DBE, including foreign bodies, diverticula, and malignancy. On an examination of the resected specimen, the Meckel’s diverticulum showed a 1-cm orifice; however, this orifice might have been difficult to detect using DBE.

At surgery, we detected a fish bone perforating the top of the Meckel’s diverticulum, but the species of fish was not identified. Most previous reports on cases of Meckel’s diverticulum perforation due to a fish bone also failed to identify the species of fish [12]. The present patient often ate fish of different species and was thus unable to recall which species might have led to his condition.

*Klebsiella pneumoniae* is the most common pathogen causing liver abscess. However, in our case, blood cultures grew *S. oralis*, and purulent fluid cultures from the liver abscess grew *S. intermedius.* These are the same genus and constitute part of the normal bacterial flora of the human mouth, nasopharynx, and GI tract. Both cultures grew the same bacterial genus, which was not *Klebsiella*, and the patient developed fish bone perforation; we therefore diagnosed the liver abscess as secondary to fish bone perforation via the portal vein. Malignant neoplasm in the lower GI has been reported to cause liver abscess via the portal vein because of (1) malignancy inhibiting immunity, (2) destruction of the intestinal wall barrier, or (3) increasing pressure in the colon causing the intestinal flora to be transported into vessels [13]. However, there have been quite a few reports fish bone perforation in the lower GI tract causing liver abscess. As shown in Table 1, several case reports have described liver abscess due to a foreign body in the GI tract directly penetrating the liver; to our knowledge, however, there is only one case report with a similar presentation to ours [13]. In that case, the patient had recurrent episodes of liver abscess and a fish bone penetrating the ileum in the right lower abdomen. Blood cultures grew *S. oralis*, and drainage cultures from the liver abscess isolated *S. intermedius*, just as in our case.

**Table 1:** Reports on liver abscess caused by fish bone perforation

Case	Year of publication	Author	Age (years)	Sex	Abscess portion	Perforation portion	How to form liver abscess
1	1990	Aoki	73	Male	Right lobe	unknown	unknown
2	1993	Tamura	61	Male	Left lobe	unknown	unknown
3	1995	Matsuzaki	56	Male	Right lobe	duodenum	directly
4	1995	Kato	73	Female	Left lobe	stomach	directly
5	1995	Mimoto	50	Male	Left lobe	stomach	directly
6	1999	Horii	61	Male	Left lobe	unknown	directly
7	2005	Oda	38	Male	Left lobe	stomach	directly
8	2006	Roki	77	Male	Left lobe	stomach	directly
9	2006	Mizunuma [3]	53	Female	Right lobe	duodenum	directly
10	2007	Nagai	54	Male	Left lobe	stomach	directly
11	2007	Kadowaki	73	Male	Left lobe	duodenum	directly
12	2008	Clarençon	64	Male	Right lobe	unknown	directly
13	2009	Matsuo	74	Female	Left lobe	stomach	directly
14	2010	Kataoka	64	Female	Left lobe	unknown	unknown
15	2011	Ohara	81	Male	Left lobe	stomach	directly
16	2013	Akimori	72	Male	Left lobe	stomach	directly
17	2014	Mukaihashi	68	Male	Left lobe	stomach	directly
18	2015	Ishikawa	63	Male	Right lobe	unknown	unknown
19	2015	Hosoi	83	Female	Left lobe	stomach	directly
**20**	**2016**	**Nagashima** [13]	**62**	**Male**	**Right lobe**	**ileum**	**via portal vein**
21	2017	Urata	75	Female	Left lobe	duodenum	directly
22	2018	Kohama	74	Male	Left lobe	unknown	directly
23	2019	Tsutsumi	41	Male	Left lobe	stomach	directly
		**Our case**	**54**	**Male**	**Right lobe**	**ileum**	**via portal vein**

To our knowledge, there has been only one case report with a similar presentation [13]; that report described a case of liver abscess secondary to fish bone perforation via the portal vein

Liver abscess is a life-threatening infection, and source control is important for the management of abdominal sepsis [14]. All abscesses more than 4 cm in size should be managed by percutaneous drainage and antibiotics [5, 6]. Treating liver abscess due to non- *K. pneumoniae* with empirical antibiotics targeting to *K. pneumoniae* has also been suggested [5]. Therefore, in our case, we performed percutaneous drainage and administered ceftriaxone and metronidazole.

Liver abscess cases due to foreign bodies require multidisciplinary treatment, so we must detect and remove the cause of the liver abscess as early as possible [15]. In addition, when exploring the cause of liver abscess, we should investigate the whole body, including the lower GI tract.

## Data Availability

Not applicable.

## Abbreviations

CT: Computed tomography
DBE: Double balloon endoscopy
GI: Gastrointestinal
IBD: Inflammatory bowel disease
